# Gene set meta-analysis with Quantitative Set Analysis for Gene Expression (QuSAGE)

**DOI:** 10.1371/journal.pcbi.1006899

**Published:** 2019-04-02

**Authors:** Hailong Meng, Gur Yaari, Christopher R. Bolen, Stefan Avey, Steven H. Kleinstein

**Affiliations:** 1 Department of Pathology, Yale School of Medicine, New Haven, Connecticut, United States of America; 2 Bioengineering Program, Faculty of Engineering, Bar-Ilan University, Ramat Gan, Israel; 3 Department of Microbiology and Immunology, Stanford University, Stanford, California, United States of America; 4 Interdepartmental Program in Computational Biology and Bioinformatics, Yale University, New Haven, Connecticut, United States of America; 5 Department of Immunobiology, Yale University School of Medicine, New Haven, Connecticut, United States of America; Johns Hopkins University, UNITED STATES

## Abstract

Small sample sizes combined with high person-to-person variability can make it difficult to detect significant gene expression changes from transcriptional profiling studies. Subtle, but coordinated, gene expression changes may be detected using gene set analysis approaches. Meta-analysis is another approach to increase the power to detect biologically relevant changes by integrating information from multiple studies. Here, we present a framework that combines both approaches and allows for meta-analysis of gene sets. QuSAGE meta-analysis extends our previously published QuSAGE framework, which offers several advantages for gene set analysis, including fully accounting for gene-gene correlations and quantifying gene set activity as a full probability density function. Application of QuSAGE meta-analysis to influenza vaccination response shows it can detect significant activity that is not apparent in individual studies.

This is a *PLOS Computational Biology* Software paper.

## Introduction

Whole-genome transcriptional profiling, using DNA microarray technology or next-generation sequencing (RNA-seq), is widely used to gain insights into disease pathophysiology and response to therapy. While it is important to identify individual genetic associations, the high level of variation between individuals due to genetic and phenotypic heterogeneity can result in inconsistent biological insights [1]. With the availability of biological annotation for known genes [2–5], the focus of gene analysis has shifted from individual genes to gene sets. Gene set analysis can be used to detect and compare the activity of pre-defined lists of genes that can be related directly to the underlying biological processes. Compared to differential expression (DE) analysis of individual genes, gene set analysis examines the cumulative effect of multiple related genes, and thus offers the possibility to detect more subtle, but coordinated, expression changes [6–10]. Despite this increased power, gene set analysis can still be limited by the small sample sizes of many current studies. Combining multiple related studies through meta-analysis offers the possibility of increased power and improved reproducibility [11]. Such studies can leverage the large and growing number of transcriptional profiling data sets available in public repositories, such as GEO [12]. However, combining information from multiple studies and performing meta-analysis at the gene set level remains challenging. Meta-Analysis of Pathway Enrichment (MAPE), including MAPE-P, MAPE-G, and MAPE-I, use maximum, minimum, or Fisher’s statistics to combine P values from each individual study for meta-analysis [13]. Instead of combining P values, MetaPath leverages a Bayesian model and was developed to perform gene set meta-analysis by simultaneously modeling gene expression data and gene set information from multiple studies [14]. Recently, Lu et al. developed iGSEA that uses an adaptive testing method for choosing either random Effects (RE) or fixed effects (FE) model to integrate gene set analysis from multiple studies [15].

We previously proposed Quantitative Set Analysis for Gene Expression (QuSAGE) [16] as a computational framework for gene set analysis. QuSAGE quantifies gene set activity with a complete probability density function (PDF), and improves power by accounting for gene-gene correlations. The QuSAGE R package is available on Bioconductor [17], and is widely used with 1554 downloads from distinct IPs in 2017. In 2015, Turner et al. extended the applicability of QuSAGE to longitudinal studies by adding functionality for general linear mixed models [18]. In this study, we further extend the applicability of QuSAGE to include meta-analysis of gene sets. QuSAGE meta-analysis was adopted by the NIH/NIAID Human Immunology Project Consortium (HIPC)–Center for Human Immunology (CHI) Signature Project Team to successfully detect baseline transcriptional predictors of influenza vaccination responses from multiple studies [19].

As an alternative gene set meta-analysis method, QuSAGE meta-analysis has several advantages: 1) It is a natural extension of QuSAGE, so it facilitates gene set meta-analysis for the large number of existing QuSAGE users, 2) QuSAGE improves power by accounting for gene-gene correlations and QuSAGE meta-analysis inherits this advantage, and 3) Since QuSAGE quantifies a gene set activity with a PDF, it is capable of performing complicated post hoc comparisons that other gene set meta-analysis methods cannot achieve easily, as we demonstrate in our case study.

### Design & implementation

**Fig 1:**
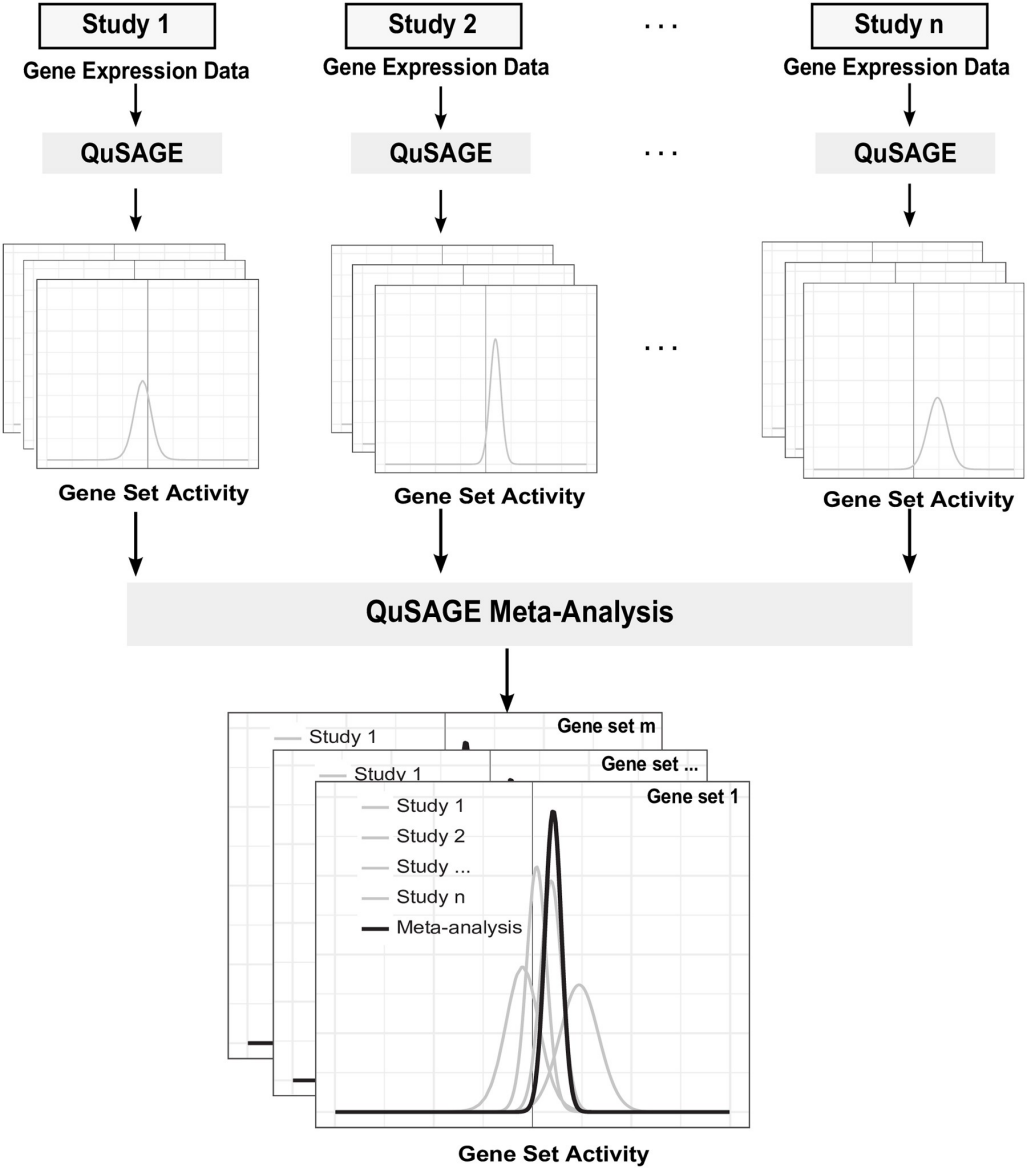
Overview of the QuSAGE meta-analysis pipeline. Gene expression data of each study is first analyzed separately by QuSAGE to produce gene set activity PDFs. Next, meta-analysis is performed through the function combinePDFs, where PDFs from each individual study are combined into a single PDF using a weighted numeric convolution algorithm. The results of QuSAGE meta-analysis can then be visualized by the function plotCombinedPDF.

QuSAGE quantifies gene set activity with a complete probability density function (PDF). The QuSAGE meta-analysis pipeline proceeds in three steps (Fig 1).

Frist, gene set analysis is performed with gene expression data separately for each individual study using QuSAGE. Differential gene expression of individual gene is quantified by a full PDF rather than a single P value. Then all PDFs of genes within the gene set of interest are combined into a single activity (PDF) using numerical convolution. The variance of the combined PDF is corrected for gene-gene correlation by calculating a variance inflation factor (VIF).

Next, the meta-analysis is performed through the function combinePDFs (Table 1). To carry out meta-analysis of *S* studies, the PDFs from each individual study are combined into a single PDF using a weighted numeric convolution algorithm [20]. The sample sizes of each study are considered as weight factors. In short, the continuous PDFs are sampled within an interval that spans their individual ranges. Each PDF is sampled by a finite number of points that is proportional to its weight. These discretized PDFs are then convoluted and the result is resampled and transformed back to the initial interval. P values and confidence intervals can be easily extracted from the resulting combined PDF.

**Table 1:** Pseudocode for QuSAGE meta-analysis.

Algorithm Pseudocode for QuSAGE Meta-Analysis		
**Input:** G gene sets and S studies		
**Output:** A combined PDF for each gene set g denoted as ${PDF}_{g}^{Meta}$		
1:	G ← number of gene sets	
2:	S ← number of studies	
3:	**for** g in 1:G **do**	
4:	**for** s in 1:S **do**	
5:	${PDF}_{gs}^{*}\leftarrow Sample({PDF}_{gs})$	// Sample in proportion to size of s
6:	${PDF}_{g}^{Meta}\leftarrow Convolution({PDF}_{g1}^{*},{PDF}_{g2}^{*},\ldots ,{PDF}_{gS}^{*})$	

Finally, the results of QuSAGE meta-analysis can be visualized by the function plotCombinedPDF.

## Results

To illustrate how QuSAGE meta-analysis works, we analyzed three influenza vaccination transcriptional profiling studies of young adults [21]. The data from these studies is available in GEO (GSE59635, GSE59654, and GSE59743) and ImmPort (SDY63, SDY404, and SDY400). The goal of the analysis was to detect gene sets associated with successful (i.e., high) antibody responses using the transcriptional response data measured from blood samples taken pre- and 7 days post-vaccination. Subjects were categorized as high-responders (HR) and low-responders (LR) based on their adjusted maximum fold change (adjMFC) from hemagglutination inhibition assay (HAI) measurements taken pre- and 28 days post-vaccination [22]. GSE59635 (SDY63) included 7 young subjects (3 LR and 4 HR); GSE59654 (SDY404) contained 13 young subjects (7 LR and 6 HR); GSE59743 (SDY400) had 15 young subjects (7 LR and 8 HR). The data and R code of this case study can be found from: https://bitbucket.org/kleinstein/qusage.

The analysis consisted of two major steps:

1. **Identify candidate vaccination response gene sets.** First, the set of 346 blood transcription modules (BTMs) described in Li et al. [4] was filtered to a smaller list of “response” sets that showed significant activity following influenza vaccination in the set of HR subjects. To define these response gene sets, QuSAGE meta-analysis was used to compare day 7 post-vaccination with pre-vaccination transcriptional profiles in HR subjects across all three studies. This analysis identified 62 response gene sets with a Benjamani-Hochberg false discovery rate (FDR) cutoff of 5%.
2. **Detect gene sets associated with successful antibody responses.** For each response gene set selected in step 1, QuSAGE was first used to carry out a two-way comparison on each study independently. A PDF reflecting the response difference between HR and LR was quantified by calculating the difference of two PDFs, one representing the temporal gene set activity in HR (day 7 vs. pre-vaccination) and the other representing LR (day 7 vs. pre-vaccination). Next, QuSAGE meta-analysis was used to combine the PDFs from the three studies into one single PDF. Statistical significance of the meta-analysis was calculated by testing whether the central tendency of the final PDF is zero using a two-sided test with 15% FDR cutoff.

**Fig 2:**
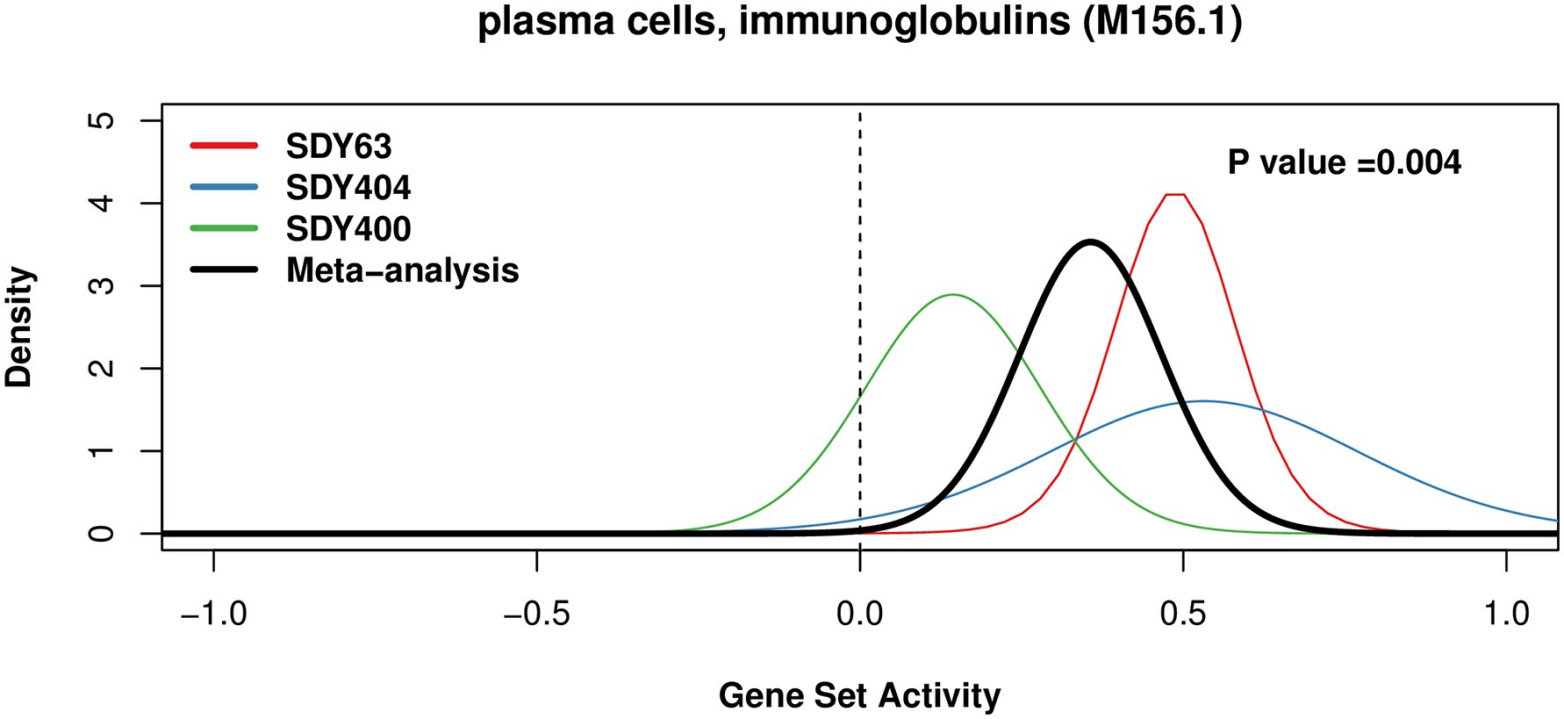
QuSAGE meta-analysis of gene set “plasma cells, immunoglobulins (M156.1)”. The differential response between HR and LR subjects was first calculated for each individual study (colored lines). QuSAGE meta-analysis was then used to combine these individual PDFs into a single meta-analysis PDF (black line).

As expected from the known biology, "plasma cells, immunoglobulins (M156.1)" was one of top-ranked gene sets from QuSAGE meta-analysis (Fig 2), and was significantly more up-regulated (day 7 vs. pre-vaccination) in HR compared to LR. In total, QuSAGE meta-analysis identified 11 gene sets associated with a successful antibody response (Table 2). In most cases (8 of 11; 73%), the QuSAGE meta-analysis of these gene sets yielded a lower P value compared with the individual studies.

**Table 2:** Nominal P values for individual studies and meta-analyses of gene sets significantly associated with successful influenza vaccination responses (FDR < 15%).

Gene Sets	SDY63	SDY404	SDY400	Meta-analysis		
				QuSAGE	Fisher	Stouffer
plasma cells, immunoglobulins (M156.1)	0.001	0.044	0.304	0.004*	0.001*	0.007
mitotic cell cycle in stimulated CD4 T cells (M4.11)	0.918	0.085	0.016	0.010*	0.038	0.028
respiratory electron transport chain (mitochondrion) (M219)	0.028	0.043	0.456	0.011*	0.020	0.038
Plasma cell surface signature (S3)	0.227	0.022	0.504	0.011*	0.062	0.069
plasma cells & B cells, immunoglobulins (M156.0)	0.002	0.139	0.326	0.011*	0.004*	0.025
respiratory electron transport chain (mitochondrion) (M216)	0.108	0.072	0.241	0.014*	0.051	0.035
transcription elongation, RNA polymerase II (M234)	0.115	0.036	0.652	0.016*	0.066	0.109
Memory B cell surface signature (S9)	0.125	0.050	0.510	0.016*	0.074	0.084
cell cycle (I) (M4.1)	0.527	0.106	0.065	0.017*	0.082	0.034
respiratory electron transport chain (mitochondrion) (M238)	0.044	0.083	0.464	0.019*	0.047	0.068
enriched in antigen presentation (I) (M71)	0.711	0.728	0.000	0.020*	0.001*	0.009
MHC-TLR7-TLR8 cluster (M146)	0.469	0.082	0.001	0.306	0.003*	0.001*

* Gene sets significantly associated with successful responses (FDR 15%), using QuSAGE, Fisher’s method or Stouffer’s method for meta-analysis,

Underlined: Gene sets where QuSAGE meta-analysis yielded lower P values compared with the individual studies

**Fig 3:**
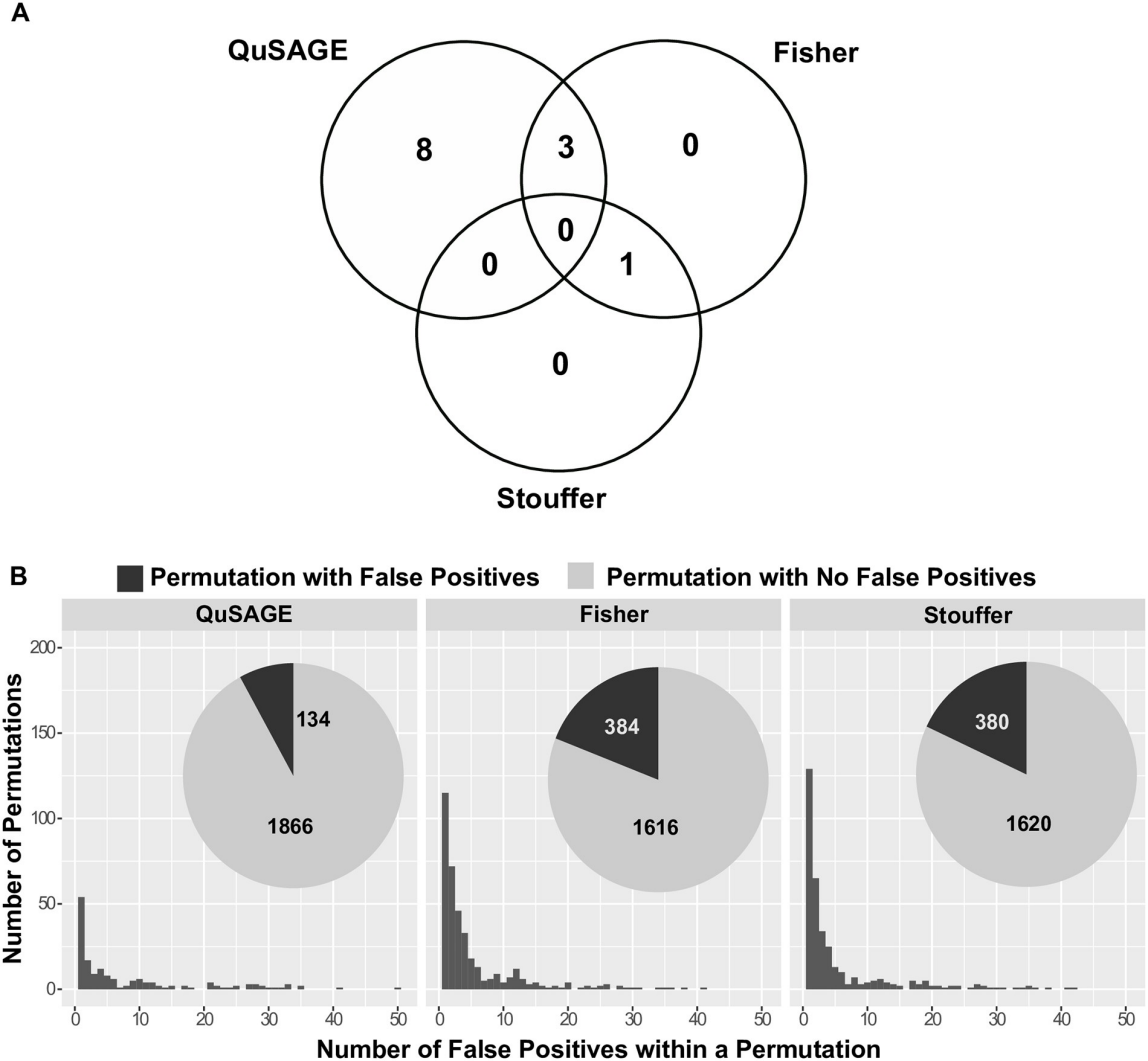
Comparison of QuSAGE with Fisher’s method and Stouffer’s method. A) Significant genes sets identified by QuSAGE meta-analysis, Fisher’s method and Stouffer’s method. Using the same FDR cutoff of 15%, QuSAGE meta-analysis, Fisher’s method and Stouffer’s method identified 11, 4 and 1 significant gene sets respectively. B) Permutation analysis of QuSAGE meta-analysis demonstrates higher specificity than Fisher’s method and Stouffer’s method. The labels of LR and HR subjects were permutated 2000 times, and meta-analysis was carried out for each of these permuted data sets. For each permutation, the number of false positive gene sets (defined at FDR < 15%) was determined for QuSAGE meta-analysis, Fisher’s method and Stouffer’s method (left, middle and right panels, respectively). The counts of permutations with and without any false positive results is indicated in the pie charts.

We next compared QuSAGE meta-analysis with other meta-analysis approaches. Existing gene set meta-analysis methods were designed to perform pairwise comparisons between two phenotypes/conditions and cannot be easily applied to the four-way comparison in our case study. For our comparative analysis, we first used Fisher’s method [23] and Stouffer’s method [24] to combine P values from QuSAGE single gene set analysis from each study and compared the results with QuSAGE meta-analysis. Using the same FDR cutoff of 15%, Fisher’s method and Stouffer’s method identified fewer gene sets than QuSAGE. Fisher’s method and Stouffer’s method identified 4 and 1 significant gene sets, respectively, including only a single gene set not found by QuSAGE (Fig 3A, Table 2). It is possible that QuSAGE meta-analysis was more sensitive, and identified additional significant gene sets, compared with Fisher’s method or Stouffer’s method at the cost of decreased specificity. To investigate the specificity of QuSAGE meta-analysis, we permutated the labels of LR and HR individuals 2000 times and applied the same meta-analyses using all three approaches. With the same FDR cutoff 15% applied to each permutation, only 134 out of 2000 permutations generated even a single false positive gene set result using QuSAGE meta-analysis; while 380 and 384 permutations produced false positives when using Fisher’s and Stouffer’s method, respectively (Fig 3B). These results suggest that QuSAGE meta-analysis is conservative and the increased number of significant gene sets identified by QuSAGE in the real data was not due to QuSAGE simply generating lower P values (i.e., QuSAGE meta-analysis is not trading off specificity for sensitivity).

**Fig 4:**
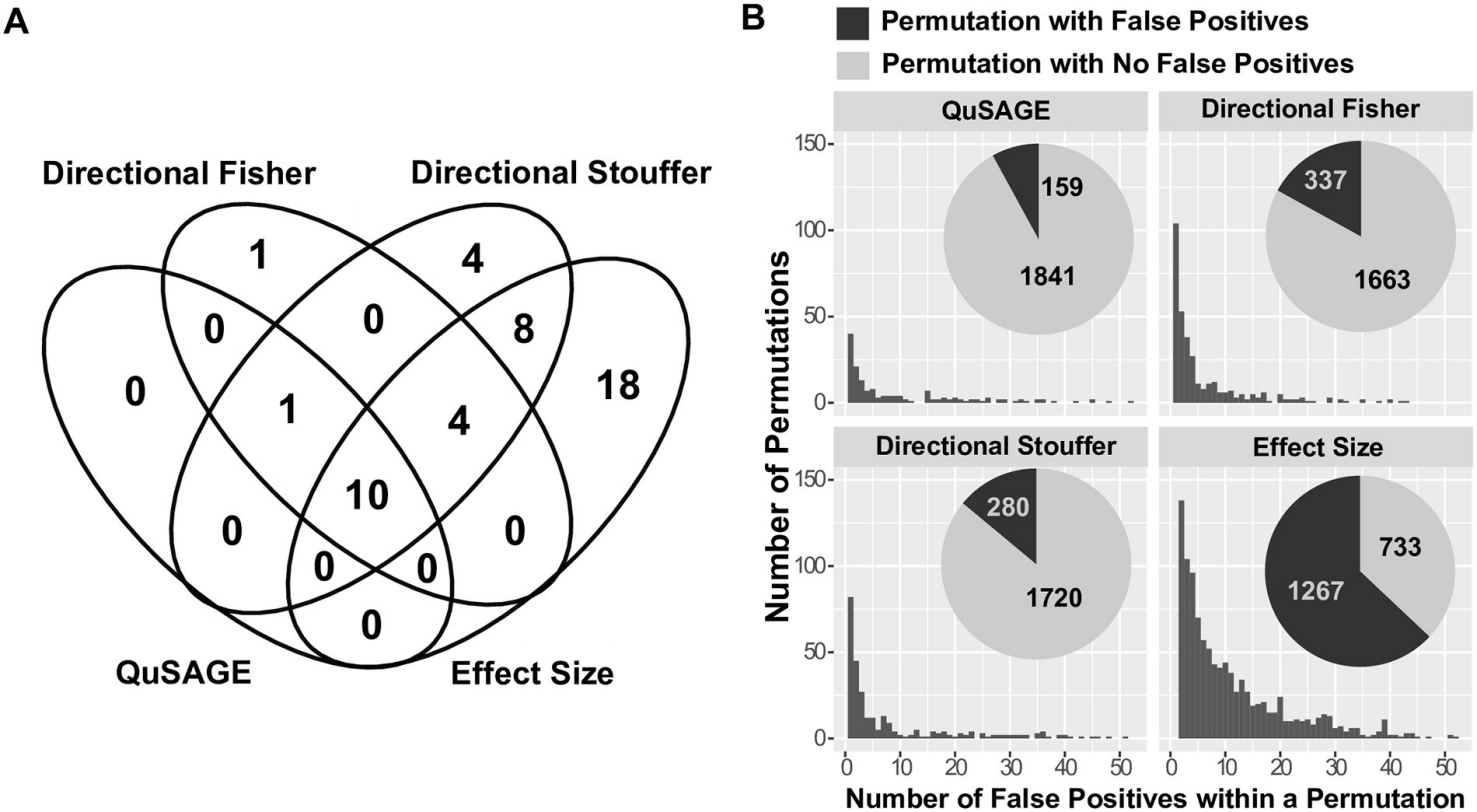
Comparison of QuSAGE with directional Fisher’s method, directional Stouffer’s method and the effect-size method. A) Significant genes sets identified by QuSAGE meta-analysis, directional Fisher’s method, directional Stouffer’s method and the effect-size method. Using the same FDR cutoff of 15%, QuSAGE meta-analysis, directional Fisher’s method, directional Stouffer’s method and the effect-size method identified 11, 16, 27 and 40 significant gene sets respectively. B) Permutation analysis of QuSAGE meta-analysis demonstrates higher specificity than directional Fisher’s method, directional Stouffer’s method and effect-size method. The labels of LR and HR subjects were permutated 2000 times, and meta-analysis was carried out for each of these permuted data sets. For each permutation, the number of false positive gene sets (defined at FDR < 15%) was determined for QuSAGE meta-analysis, directional Fisher’s method, directional Stouffer’s method and the effect-size method. The counts of permutations with and without any false positive results is indicated in the pie charts.

However, a limitation of Fisher’s method and Stouffer’s method is that neither accounts for the direction of gene set activity (e.g., higher in HR vs. higher in LR), but simply combines the resulting P values from each individual study. As a consequence, low P values may be produced by cases where the change for the individual studies is significant but in different directions, leading to false positives. To account for the directionality of gene set activity differences when applying Fisher’s method and Stouffer’s method, we carried out a three-step analysis, which were referred to directional Fisher’s method and directional Stouffer’s method. First, separate one-tailed tests were carried out for each study to test for (1) higher gene set activity in HR, and (2) higher gene set activity in LR. In this way, lower P values in each type of one-tailed test, have a consistent meaning. Second, in the meta-analysis, Fisher’s method or Stouffer’s method was applied to the set of P values from each type of one-tailed test to generate a combined P values. Third, the final P value of the meta-analysis was the smaller of the two combined P value from each of the one-tailed tests, corrected by multiplying by 2. We also tested another popular meta-analysis method in which effect sizes (Hedges’ g) are calculated for every gene set in each study separately and then combined using linear (mixed-effects) models (implemented in the rma() function from the metafor R package, and hereafter referred to as the “effect-size” method) [25]. Using the same FDR cutoff of 15%, directional Fisher’s method, Stouffer’s method and the effect-size method identified 16, 27 and 40 significant gene sets respectively (S1 Table). All 11 gene sets detected by QuSAGE meta-analysis were found by directional Fisher’s method and directional Stouffer’s method, and 10 of the 11 gene sets were found by the effect-size method, suggesting a high level of confidence in the QuSAGE results (Fig 4A). To quantify the specificity of the three approaches, we permutated the labels of LR and HR individuals 2000 times and applied the same meta-analyses on each permuted data set. With the same FDR cutoff 15% applied to each permutation, QuSAGE meta-analysis generated false positive results in only 8% (159 out of 2000) of the permutations (Fig 4B). In contrast, directional Fisher’s method, directional Stouffer’s method and the effect-size method generated at least one false positive gene set in 17%, 14% and 63% (337, 280 and 1267 out of 2000) of the permutations, respectively (Fig 4B).This higher false positive rate may account, at least partially, for the additional gene sets identified by directional Fisher’s method, directional Stouffer’s method and the effect-size method. Overall, the results on this case study show that QuSAGE meta-analysis is comparable with existing methods, but has better specificity.

In this study, we describe an extension of QuSAGE to enable meta-analysis of gene sets. Instead of summarizing P values, QuSAGE integrates gene set activity and estimates a full PDF of activity across multiple studies, thus easing the process of post hoc comparisons. Furthermore, by integrating information from a larger pool of samples, QuSAGE meta-analysis increases the power of analysis, and allows detection of biologically-relevant gene sets that would not be detectable in single studies. Existing common meta-analysis methods, such as Fisher’s method, Stouffer’s method, or the effect-size method, are limited by the fact that the gene set activity from each study is represented by a single P value (Stouffer weighs P values by sample size from each study) or a single statistic (effect size). However, QuSAGE describes the gene set activity using a PDF and the meta-analysis of QuSAGE fully takes the advantage of the richer information provided from PDFs. QuSAGE meta-analysis combines PDFs from multiple studies using a weighted numeric convolution algorithm, and thus implicitly considers not only the differences but also directions and confidence intervals of gene set activities, leading to a more accurate estimation of combined gene set activity. The QuSAGE algorithm is also computationally efficient. It took totally only 4 minutes to run the whole case study in our manuscript on a single PC with a 2.80GHz Intel Core i7 CPU and 16G memory. Our case study suggests that QuSAGE is comparable or better than the commonly used Fisher and Stouffer methods. In the future, performing comparisons of QuSAGE with other existing meta-analysis methods [13–15, 26]would be desirable.

### Availability and Future Directions

The QuSAGE R package is available in Bioconductor and can be accessed from: http://bioconductor.org/packages/release/bioc/html/qusage.html. QuSAGE meta-analysis is included in version 2.12.0 or later. The data and R code of this case study can be found from: https://bitbucket.org/kleinstein/qusage.

## Supporting information

S1 Table Nominal P values of gene sets significantly associated with successful influenza vaccination responses from four meta-analysis approaches. (DOCX)
