# Supplementary material for: Gene set meta-analysis with Quantitative Set Analysis for Gene Expression (QuSAGE)
Source: PLoS Comput Biol. 2019 Apr 2;15(4):e1006899. doi: 10.1371/journal.pcbi.1006899 (PMC6461294; doi:10.1371/journal.pcbi.1006899)
Supplement: S1 Table — (DOCX) [file pcbi.1006899.s001.DOCX]

**Supplementary Table 1.** Nominal P values of gene sets significantly associated with successful influenza vaccination responses from four meta-analysis approaches (FDR < 15%).

| Gene Set | QuSAGE Meta-analysis | Directional Fisher | Directional Stouffer | Effect Size |
| --- | --- | --- | --- | --- |
| cell cycle (I) (M4.1) | 0.017* | 0.082* | 0.034* | 0.001* |
| mitotic cell cycle in stimulated CD4 T cells (M4.11) | 0.010* | 0.038* | 0.028* | 0.011* |
| plasma cells & B cells, immunoglobulins (M156.0) | 0.011* | 0.004* | 0.025* | 0.003* |
| plasma cells, immunoglobulins (M156.1) | 0.004* | 0.001* | 0.007* | 0.013* |
| respiratory electron transport chain (mitochondrion) (M216) | 0.014* | 0.051* | 0.035* | 0.000* |
| respiratory electron transport chain (mitochondrion) (M219) | 0.011* | 0.020* | 0.038* | 0.002* |
| transcription elongation, RNA polymerase II (M234) | 0.016* | 0.066* | 0.109* | 0.015* |
| respiratory electron transport chain (mitochondrion) (M238) | 0.019* | 0.047* | 0.068* | 0.001* |
| Plasma cell surface signature (S3) | 0.011* | 0.062* | 0.069* | 0.017* |
| Memory B cell surface signature (S9) | 0.016* | 0.074* | 0.084* | 0.005* |
| enriched in antigen presentation (I) (M71) | 0.020* | 0.001* | 0.009* | 0.282 |
| mitotic cell cycle in stimulated CD4 T cells (M4.5) | 0.029 | 0.031* | 0.043* | 0.034* |
| regulation of antigen presentation and immune response (M5.0) | 0.082 | 0.037* | 0.026* | 0.058* |
| enriched in antigen presentation (III) (M95.1) | 0.049 | 0.041* | 0.026* | 0.081* |
| respiratory electron transport chain (mitochondrion) (M231) | 0.036 | 0.061* | 0.060* | 0.000* |
| MHC-TLR7-TLR8 cluster (M146) | 0.202 | 0.003* | 0.001 | 0.618 |
| PLK1 signaling events (M4.2) | 0.052 | 0.230 | 0.126* | 0.000* |
| C-MYC transcriptional network (M4.12) | 0.056 | 0.253 | 0.157* | 0.000* |
| mitotic cell division (M6) | 0.043 | 0.178 | 0.089* | 0.000* |
| RA, WNT, CSF receptors network (monocyte) (M23) | 0.085 | 0.252 | 0.134* | 0.014* |
| suppression of MAPK signaling (M56) | 0.077 | 0.280 | 0.172* | 0.078* |
| proinflammatory dendritic cell, myeloid cell response (M86.1) | 0.085 | 0.280 | 0.151* | 0.001* |
| putative targets of PAX3 (M89.1) | 0.083 | 0.290 | 0.167* | 0.027* |
| growth factor induced, enriched in nuclear receptor subfamily 4 (M94) | 0.081 | 0.295 | 0.160* | 0.005* |
| mismatch repair (I) (M22.0) | 0.135 | 0.122 | 0.085* | 0.327 |
| inflammasome receptors and signaling (M53) | 0.074 | 0.158 | 0.153* | 0.210 |
| putative targets of PAX3 (M89.0) | 0.074 | 0.272 | 0.336* | 0.142 |
| enriched in activated dendritic cells (I) (M119) | 0.067 | 0.075 | 0.075* | 0.154 |
| targets of FOSL1/2 (M0) | 0.294 | 0.676 | 0.495 | 0.044* |
| AP-1 transcription factor network (M20) | 0.227 | 0.623 | 0.448 | 0.004* |
| mismatch repair (II) (M22.1) | 0.115 | 0.340 | 0.195 | 0.006* |
| proinflammatory cytokines and chemokines (M29) | 0.325 | 0.455 | 0.279 | 0.039* |
| cell cycle and growth arrest (M31) | 0.195 | 0.566 | 0.383 | 0.017* |
| signaling in T cells (I) (M35.0) | 0.159 | 0.428 | 0.378 | 0.013* |
| chemokines and receptors (M38) | 0.354 | 0.531 | 0.366 | 0.006* |
| RIG-1 like receptor signaling (M68) | 0.283 | 0.644 | 0.536 | 0.067* |
| chemokines and inflammatory molecules in myeloid cells (M86.0) | 0.116 | 0.418 | 0.253 | 0.009* |
| enriched in antigen presentation (II) (M95.0) | 0.196 | 0.450 | 0.382 | 0.005* |
| TBA (M102) | 0.289 | 0.504 | 0.562 | 0.063* |
| enriched for TF motif TTCNRGNNNNTTC (M172) | 0.213 | 0.469 | 0.352 | 0.001* |
| TBA (M190) | 0.171 | 0.503 | 0.340 | 0.010* |
| chaperonin mediated protein folding (I) (M204.0) | 0.124 | 0.297 | 0.182 | 0.001* |
| purine nucleotide biosynthesis (M212) | 0.241 | 0.540 | 0.515 | 0.038* |
| enriched for TF motif TNCATNTCCYR (M232) | 0.633 | 0.818 | 0.673 | 0.074* |
| TBA (M242) | 0.132 | 0.420 | 0.535 | 0.035* |
| TBA (M243) | 0.045 | 0.186 | 0.332 | 0.046* |

*: Gene sets significantly associated with successful influenza vaccination responses (FDR < 15%).
